# Supplementary figures and images for: Regulation of olfactory-based sex behaviors in the silkworm by genes in the sex-determination cascade
Source: PLoS Genet. 2020 Jun 10;16(6):e1008622. doi: 10.1371/journal.pgen.1008622 (PMC7307793; doi:10.1371/journal.pgen.1008622)

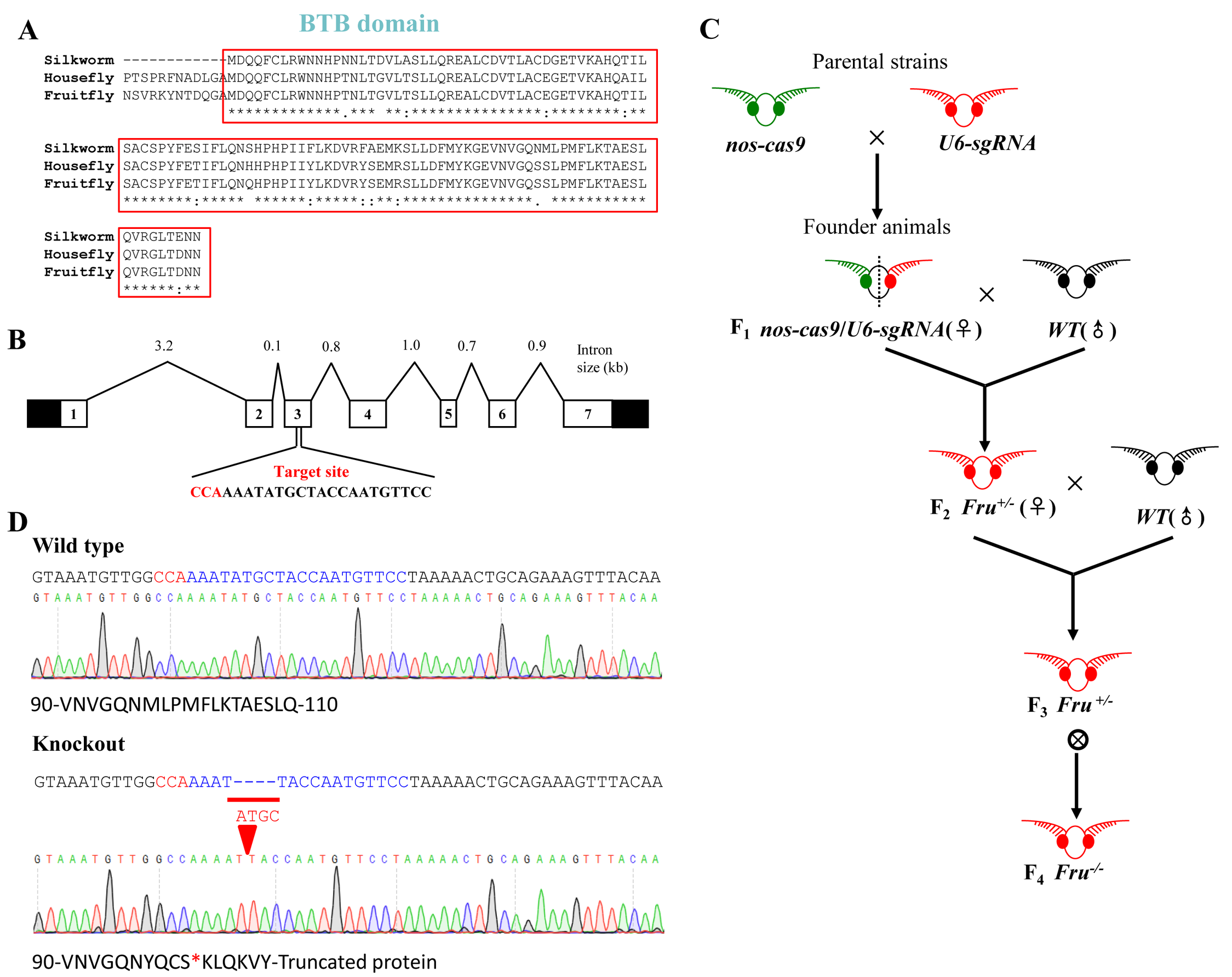

Supplement: S1 Fig — (A) The BmFRU protein, which contains the BTB domain conserved in dipteran insects, D. melanogaster and M. domestica. (B) Schematic representation of the exon/intron boundaries of the Bmfru gene. Exons are shown as boxes. Untranslated regions are shown as black boxes and coding regions as open boxes. Thin lines represent the introns and numbers are the lengths in kilobase pairs (kb). Target site locations are noted and PAM sequences are shown in red. (C) Crossing scheme to produce homozygous mutations. The binary transgenic CRISPR/Cas9 system in this study contains two lines, one of which contains the full Cas9 ORF driven by the nanos (nos) promoter, and the other contains a U6 promoter-driven sgRNA. These two lines also encode the reporter genes EGFP and DsRed2, respectively. The two transgenic lines were crossed to produce founder animals that express both Cas9 and Bmfru sgRNAs. The founder female silkworms were backcrossed with wild-types to obtain heterozygous offspring (F2, Fru+/-). F2 heterozygous mutant females were individually crossed with wild-type males to obtain distinct F3 heterozygous lines. F3 moths heterozygous for the mutations were sib-mated to generate independent lines of homozygous animals (F4, Fru-/-). (D) Homozygous mutations confirmed by sequence analysis. The targeting sequence is shown in blue and the PAM sequence in red. The deleted base pairs (bp), ATGC, are indicated by the broken line. (TIF) [file pgen.1008622.s001.tif]

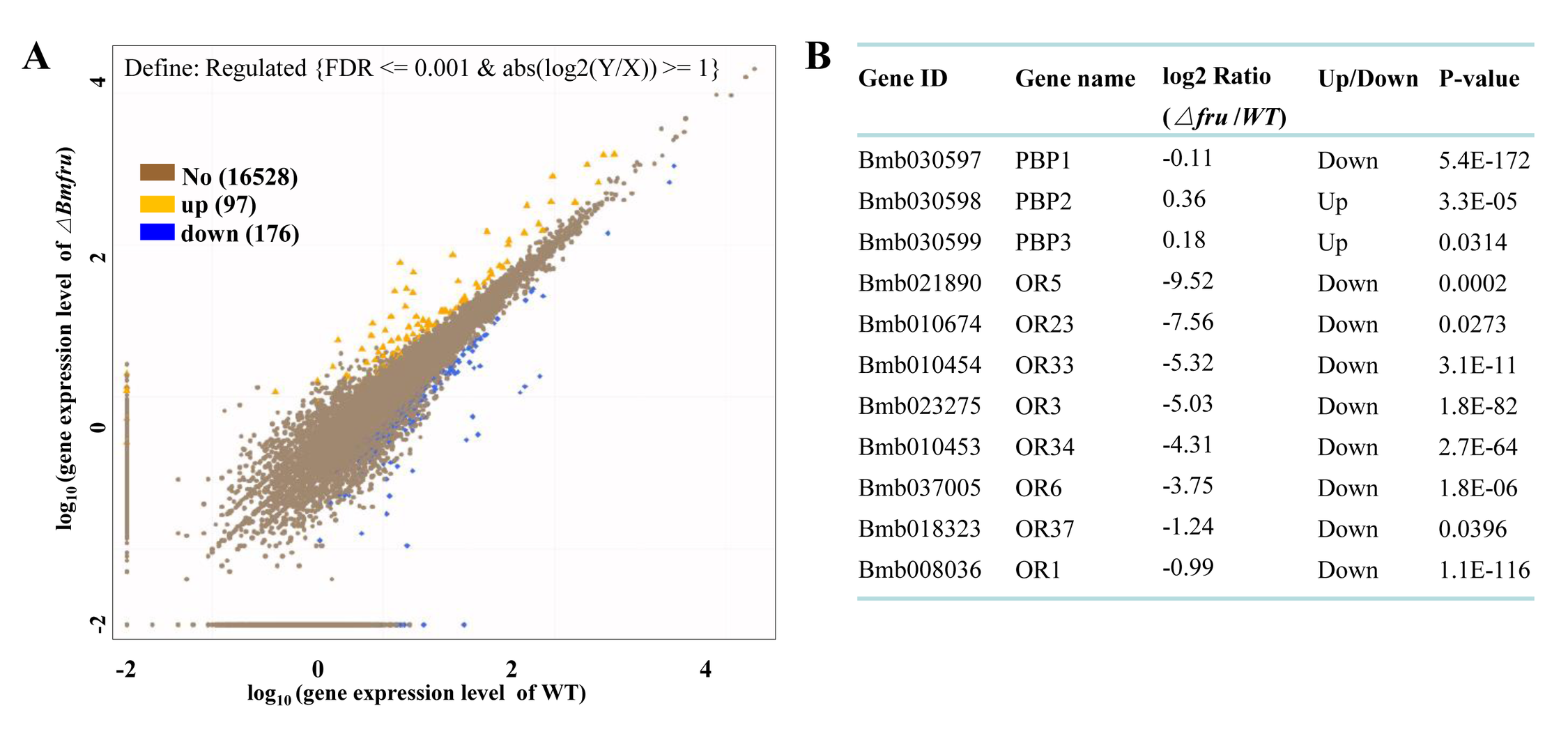

Supplement: S2 Fig — (A) Plot of significantly differentially expressed genes in 10 mixed Bmfru mutant male antennas compared to 10 mixed WT adult male antennas. False discovery rate (FDR) was used to determine the threshold of p values in multiple tests. We use FDR ≤ 0.001 and the absolute value of log2Ratio ≥ 1 as thresholds to determine significant differences in gene expression. Yellow represents up-regulated genes, blue represents down-regulated genes, and gray represents genes without significant differences. (B) Olfactory sensory system genes with changes significant at p<0.05. (TIF) [file pgen.1008622.s002.tif]
