# Supplementary material for: Regulation of olfactory-based sex behaviors in the silkworm by genes in the sex-determination cascade
Source: PLoS Genet. 2020 Jun 10;16(6):e1008622. doi: 10.1371/journal.pgen.1008622 (PMC7307793; doi:10.1371/journal.pgen.1008622)
Supplement: S1 Text — (DOCX) [file pgen.1008622.s008.docx]

>Silkworm

MDQQFCLRWNNHPNNLTDVLASLLQREALCDVTLACDGETVKAHQTILSACSPYFESIFLQNSHPHPIIFLKDVRFAEMKSLLDFMYKGEVNVGQNMLPMFLKTAESLQVRGLTENNTLNPKSEERSTPVVGAENLSRAEFATPPAAHCGPPVGPAPPPAHPAHAQHPSHPAQHVPLEKRRRKNSTLPRDDIDNNLYRHYDVPVKSGKCSTGSGSEPSTPPPAHTPHGAQRLGTRSPLVKQEPDSPYMPHVPHPSPFEQQHLQNIGVTDMASILSQPPINNDCNESEPSLPPHPDQTDTIDGDNFGVDENKKPWEPFNNKLSNVENLVKSFKLALTQRPFSGPESCNVCGKLVNNLKKHMKSHNPEQHKCPLCPIILTRADNLKRHLRMKHCSVLSSDNLSNIQKKCGLT

>Housefly

MMTTSQHFFNNPYAMFHGPPPKMGPPESPHNTYALDLHTTTKPRTLDREERPPPYTPPPPPTSPRFNADLGAMDQQFCLRWNNHPTNLTGVLTSLLQREALCDVTLACEGETVKAHQAILSACSPYFETIFLQNHHPHPIIYLKDVRYSEMRSLLDFMYKGEVNVGQSSLPMFLKTAESLQVRGLTDNNNLNYPSELDKHRDADISSPTGRTSYGAGGGAGGPGLGMRGERESRDRGRGEMRDDLHSHRSSSSLSERSSATAAAVAAAVAAASGNASLQSAAATLGLTGGERSPSVGSASAAAAAVAAVVAAAAGRSASADVLNSRGDAGSDRGSDRGNDNSVCGGVDRGGGIDERRDDLGQIDYSNQSKRDRDREVSTTPEHIISNKRRRKNSSNCDNLLTSTPNANVQDRHYAQDSQAPSNFKSSPVPKSSTGGGGGGNTSETEDSGGRRDSPLSASALSGGGNVNASSGGMGLNQSLSIKQELMDAQQQQQREHHVSLPPEYLPPGALKHSEDMASLLSSHSMQAADSREDHNDAKQLPFDQSDNIDGEIIEGGGDGDGEGVVPMRASGEDSDGNGKGCGNAADLQHHYMHNVQRAGKLNDLNDNAEGGGGRNSAEDDDDDDSNGISAESRVHHQQQQSHCRRILSRHDPDGGHYTDVTEVAVDVDNEEINMTNSSYNCQYKSDDLSLTKIRCHQRDVYGHHQEPSHLDHASQQPSTSHRHMHHHHHHPHHHSLHESHVPHQQHVAAAPQSVINLGRCGSSSLDTLVAAEAAAAALSPTASSTSSASLQHQQHMYALQQHHHHGQEQQQQSSLHHHHHHASQHGSGAQHVANYHHGHHPKLHPCTNTSMVSSTAMQSAAHDSEHLTNTSSSTSSTSASSAAAAAAAAAAANRRDHNIDYSLLFVQLSGTLPTLYRCVSCNKIVSNRWHHANIHRPQSHECPVCGQKFTRRDNMKAHCKIKHADIKDRFFSHYVHM

>Fruitfly

MLQKNSVRKYNTDQGAMDQQFCLRWNNHPTNLTGVLTSLLQREALCDVTLACEGETVKAHQTILSACSPYFETIFLQNQHPHPIIYLKDVRYSEMRSLLDFMYKGEVNVGQSSLPMFLKTAESLQVRGLTDNNNLNYRSDCDKLRDSAASSPTGRGPSNYTGGLGGAGGVADAMRESRDSLRSRCERDLRDELTQRSSSSMSERSSAAAAAAAAAAAVAAAGGNVNAAAVALGLTTPTGGERSPSVGSASAAAAAAAVAAAVAAAANRSASADGCSDRGSERGTLERTDSRDDLLQLDYSNKDNNNSNSSSTGGNNNNNNNNNNNSSSNNNNSSSNRERNNSGERERERERERERDRDRELSTTPVEQLSSSKRRRKNSSSNCDNSLSSSHQDRHYPQDSQANFKSSPVPKTGGSTSESEDAGGRHDSPLSMTTSVHLGGGGGNVGAASALSGLSQSLSIKQELMDAQQQQQHREHHVALPPDYLPSAALKLHAEDMSTLLTQHALQAADARDEHNDAKQLQLDQTDNIDGRVKCFNIKHDRHPDRELDRNHREHDDDPGVIEEVVVDHVREMEAGNEHDPEEMKEAAYHATPPKYRRAVVYAPPHPDEEAASGSGSDIYVDGGYNCEYKCKELNMRAIRCSRQQHMMSHYSPHHPHHRSLIDCPAEAAYSPPVANNQAYLASNGAVQQLDLSTYHGHANHQLHQHPPSATHPSHSQSSPHYPSASGAGAGAGSVSVSIAGSASGSATSAPASVATSAVSPQPSSSSTGSTSSAAAVAAAAAAAANRRDHNIDYSTLFVQLSGTLPTLYRCVSCNKIVSNRWHHANIHRPQSHECPVCGQKFTRRDNMKAHCKIKHADIKDRFFSHYVHM
